# Prevention of hospital-acquired and central line-associated bloodstream infections in the intensive care unit through chlorhexidine gluconate washcloth bathing: a systematic review and meta-analysis

**DOI:** 10.1186/2197-425X-3-S1-A446

**Published:** 2015-10-01

**Authors:** EP Afonso, K Blot, S Blot

**Affiliations:** Cambridge University Hospitals - Rosie Hospital, Neonatal Intensive Care Unit, Cambridge, United Kingdom; Faculty of Medicine and Health Sciences, Ghent University, Ghent, Belgium; Dept. of Internal Medicine, Faculty of Medicine and Health Science, Ghent University, Ghent, Belgium Belgium; Burns Trauma and Critical Care Research Centre, The University of Queensland, Brisbane, Australia

## Background

Bloodstream Infection and Central Line-Associated Bloodstream Infection (BSI/CLABSI) in Intensive Care Units (ICUs) are associated with clinical and economic burden. Chlorhexidine gluconate body washing with washcloths (CHG-WC) has been described as potentially effective towards reducing the spread of infection. Current systematized evidence has not fully ascertained the impact of CHG-WC in bacteremia within the ICU. We have systematically assessed the evidence on the effectiveness of CHG-WC in reducing total HABSI/CLABSI in the ICU.

## Objectives

To systematically review the effect of CHG-WC on total HABSI/CLABSI risk in the ICU.

## Methods

Medline, EMBASE, Cochrane library (CENTRAL) and Web of science databases were searched using variations of key terms Chlorhexidine washcloths, ICU, BSI and CLABSI. Eligible trials implemented CHG-WC in ICUs using a randomized crossover study design and reported total HABSI and CLABSI rates per patient days. Methodological quality of studies was assessed using Cochrane Risk of Bias assessment tool. Random effects meta-analysis calculated odds ratios (OR) and 95% confidence intervals (CI) for total HABSI and CLABSI rates between control (regular washcloth bathing) and treatment groups (CHG-WC). Results of 4 eligible studies were included in the final analysis.

## Results

All 4 eligible studies were large randomized control trials encompassing 706 events in 22850 patients from 15 adult and 10 paediatric ICUs. Three studies describe a reduction in total HABSI/CLABSI on patients who received CHG-WC bathing. In one study this reduction is not significant.

## Conclusions

Current data suggests that the use of CHG-WC may be associated with an overall risk reduction of total HABSI and CLABSI, in adult/paediatric ICUs. However, conflicting data remains from the most recent randomized controlled trial, and even though this can be related to discrepancies in study design and outcome reporting, more trials are needed in order to ascertain the usefulness of CHG-WC bathing in the ICU.

**Figure 1:**
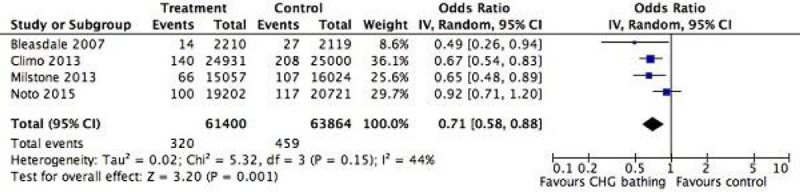
**Overall impact of CHG-WC in HABS/CLABSI.**

**Figure 2:**
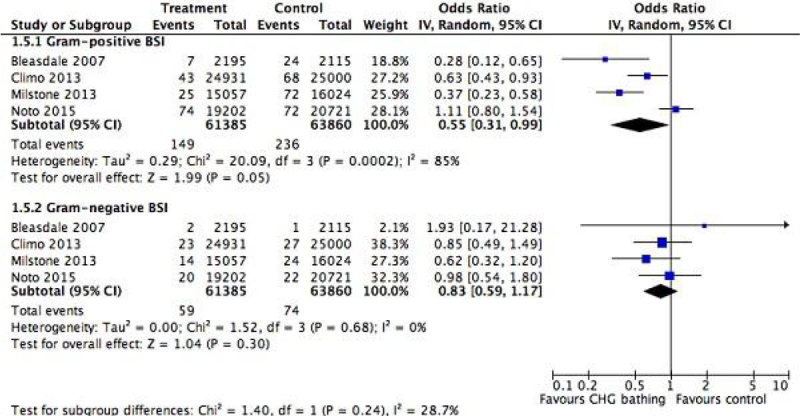
**Subgroup analysis.**
